# Primary intradural extramedullary spinal mesenchymal chondrosarcoma: case report and literature review

**DOI:** 10.1186/s12891-019-2799-2

**Published:** 2019-09-04

**Authors:** Chih-Wei Chen, I-Hsin Chen, Ming-Hsiao Hu, Jen-Chieh Lee, Hsuan-Ying Huang, Ruey-Long Hong, Shu-Hua Yang

**Affiliations:** 1 Department of Orthopedics, National Taiwan University College of Medicine and National Taiwan University Hospital, No.7, Chungshan South Road, 10002 Taipei, Taiwan; 2 Department of Orthopedics, National Taiwan University Hospital Hsin Chu Branch, Hsin Chu, Taiwan; 3 Graduate Institute and Department of Pathology, National Taiwan University College of Medicine and National Taiwan University Hospital, Taipei, Taiwan; 4 Department of Pathology, Kaohsiung Chang Gung Memorial Hospital and Chang Gung University College of Medicine, Kaohsiung, Taiwan; 5 Department of Oncology, National Taiwan University College of Medicine and National Taiwan University Hospital, Taipei, Taiwan

**Keywords:** Intradural, Extramedullary, Mesenchymal chondrosarcoma, Spine

## Abstract

**Background:**

Mesenchymal chondrosarcoma (MCS) is a rare malignant variant of chondrosarcoma with a high tendency of recurrence and metastasis. Intradural extramedullary spinal MCS is exceedingly rare and usually found in pediatric patients. Herein, we present an elderly patient with primary intradural extramedullary spinal MCS. Relevant literatures are reviewed to disclose characteristics of intradural extramedullary spinal MCS.

**Case presentation:**

A 64-year-old female presented with urinary difficulty and tightness of upper back preceding progressive weakness of right lower extremity. Magnetic resonance imaging revealed an intradural extramedullary tumor at the level of 3rd thoracic vertebra. This patient underwent total tumor resection and then received adjuvant radiotherapy. Histopathological examination showed that the tumor composed of spindle and round cells with high nucleocytoplasmic ratio accompanied by scattered eosinophilic chondroid matrix. Along with immunohistochemical findings and the existence of *HEY1-NCOA2* fusion transcript, the diagnosis of MCS was confirmed. Neurologic deficit recovered nearly completely after surgery. No evidence of local recurrence or distant metastasis was found 5 years after treatments. Including the current case, a total of 18 cases have been reported in the literature with only one case with local recurrence and one case of mortality. The current case was the eldest patient diagnosed with primary intraspinal MCS in the literature.

**Conclusions:**

MCS rarely appears in the intradural space of the spine. In contrast to classic MCS, treatment outcome of primary intradural extramedullary spinal MCS is usually excellent as total tumor resection is commonly achievable. Adjuvant radiotherapy may reduce local recurrence and chemotherapy may be associated with fewer recurrences especially for unresectable tumors.

**Electronic supplementary material:**

The online version of this article (10.1186/s12891-019-2799-2) contains supplementary material, which is available to authorized users.

## Background

Mesenchymal chondrosarcoma (MCS) is a rare malignant variant of chondrosarcoma whose incidence accounts for 0.2–0.7% of all malignant bone tumors or 3–10% of chondrosarcoma [1]. Even though a majority of these tumors are believed to be originated from bone, there is a considerable percentage around 33–50% that they can be detected in the extra-skeletal sites. Extra-skeletal MCSs most often involve the brain and meninges, occasionally intraspinal region [2]. Among them, intradural extramedullary MCS is exceedingly rare, which only has been described in sparse case reports with variable clinical traits. Recently, a novel fusion gene, *HEY1-NCOA2*, has been identified and adopted to confirm the diagnosis of MCS [3]. On the other hand, the vast majority of intradural extramedullary spinal tumors of adults are benign, in which the most common histological types are nerve sheath tumors, including schwannomas and neurofibromas, meningiomas, or ependymomas of the filum terminale [4]. MCS is a rare pathology involving the intradural extramedullary space of the spine. In this report, we document an adult case with primary intradural extramedullary MCS in the thoracic spine treated with surgical resection and adjuvant radiotherapy. Preoperative neurologic deficit recovered nearly completely after surgery. No local recurrence or distant metastasis was found 5 years after surgery.

## Case presentation

**Fig. 1:**
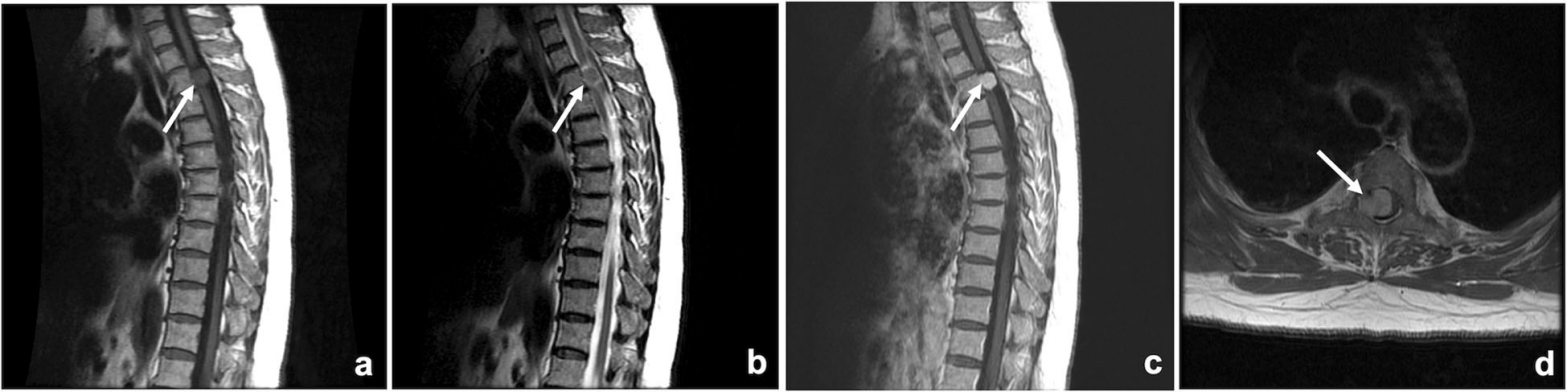
Preoperative MR images. **a** Sagittal T1-weighted image (T1WI), **b** Sagittal T2-weighted image (T2WI), **c** Sagittal T1WI with gadolinium enhancement, and **d** Axial T1WI with gadolinium enhancement. An intradural extrameduallary mass located at T3 level (arrows) and was characterized by intermediate signal intensity at T1WI, mildly increased signal intensity at T2WI and evident enhancement after gadolinium administration. Severe spinal cord compression by the tumor was shown (**d**)

A 64-year-old woman initially experienced urinary difficulty and felt tightness at upper back. Weakness of right lower extremity developed 2 months later and rapidly progressed to inability of ambulation in following 2 weeks. She visited authors’ institute with presentation of monoplegia of right lower extremity and asymmetrically decreased response to light touch and pain below nipples, more prominent on the left side. Hyper-reflexia and positive Babinski sign at bilateral lower extremities were also noted. The results of routine blood tests were unremarkable and the patient had no family history of cancer or genetic disease. As the clinical manifestation was compatible with Brown-Séquard syndrome, a compressive lesion to spinal cord at the thoracic spine was suspected. Magnetic resonance imaging (MRI) revealed an intradural extramedullary mass at the level of 3rd thoracic (T3) vertebra with severe compression to the spinal cord (Fig. 1). The tumor was about 1.5 cm in size and characterized by intermediate signal intensity at T1-weighted images with mildly increased signal intensity at T2-weighted images and evident enhancement after the gadolinium administration. There was no bony involvement or other lesion found in radiologic assessments.

The patient received surgical treatment for removal of the tumor mass on the next day after MRI study. During the surgery, the posterior surface of dural sac was exposed after total laminectomy from T2 to T4 vertebrae and then the intradural space was accessed through longitudinal opening of the dura mater. Grossly, the tumor sized 2 × 1.5 × 1.5 cm and was firm, reddish, lobulated, and hypervascular. The mass was attached to the inner surface of dura mater with no involvement of arachnoid or spinal cord. The tumor was removed *en bloc* after detached from dura mater. Posterior instrumentation and fusion were performed to prevent post-laminectomy kyphosis.

**Fig. 2:**
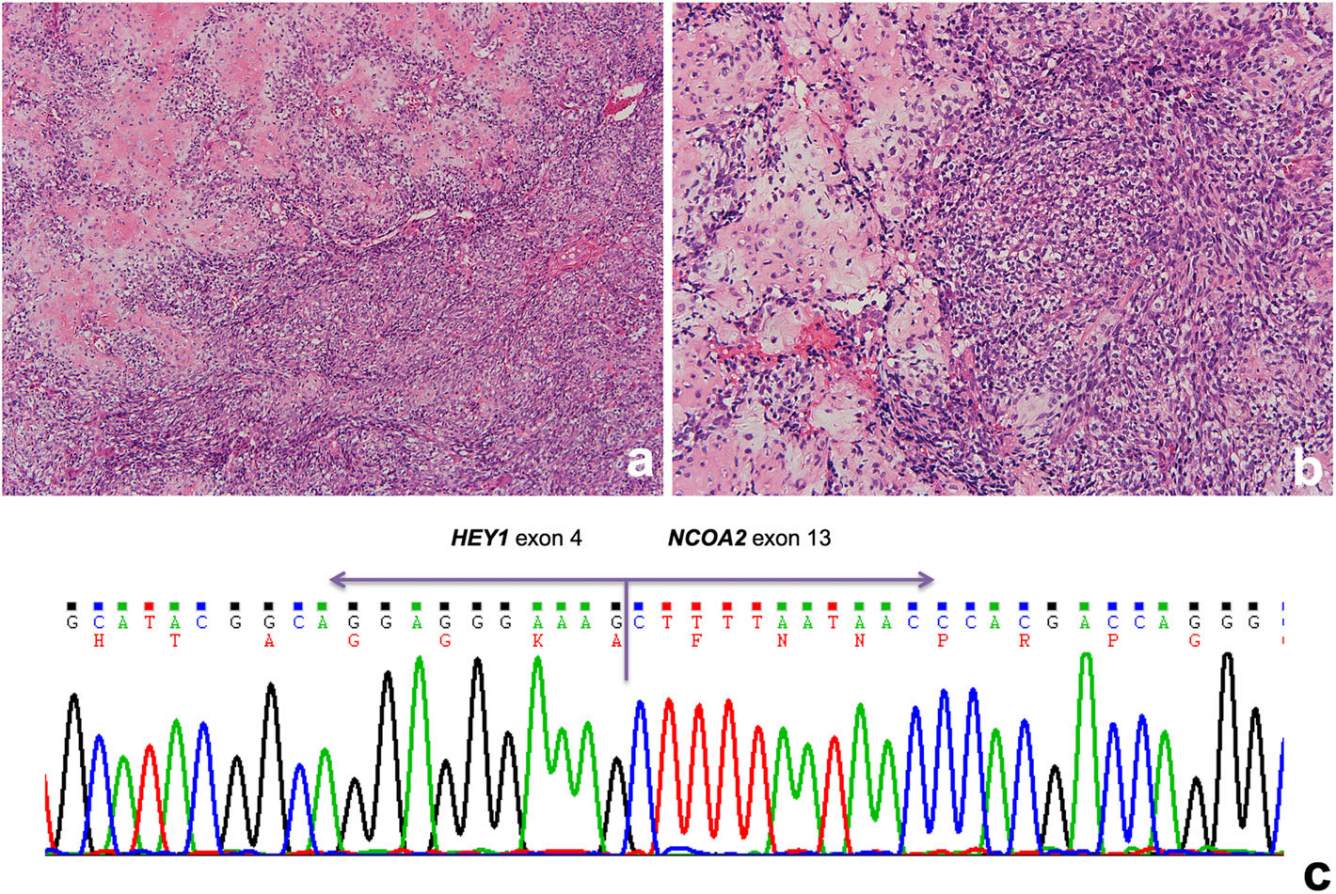
**a** At the × 100 magnification, the tumor is hypercellular and composed of spindle and round cells with a high nucleocytoplasmic ratio accompanied by scattered eosinophilic chondroid matrix. **b** At the × 200 magnification, the tumor cells have ovoid to round nuclei and inconspicuous cytoplasm, arranged in vague fascicles. Note the eosinophilic chondroid matrix (left field). **c** RT-PCR confirmed the presence of *HEY1-NCOA2* fusion

Histological examination revealed that the tumor was hypercellular and composed of spindle and round cells with a high nucleocytoplasmic ratio accompanied by scattered eosinophilic chondroid matrix (Fig. 2a). The tumor cells had ovoid to round nuclei and inconspicuous cytoplasm, arranged in vague fascicles (Fig. 2b). As for immunohistochemistry, the tumor cells were positive for CD99, desmin (especially on the chondrocyte-like cells) and CDK4 (focally), while negative for S100 protein, CK (AE1/AE3), CD34, MDM2, and myogenin. In addition, reverse transcription polymerase chain reaction (RT-PCR) was positive for *HEY1-NCOA2* fusion transcript (Fig. 2c). Collectively, a mesenchymal chondrosarcoma of possible meningeal origin was diagnosed.

The postoperative course was rather smooth with muscle power of right lower limb improved rapidly and completely after surgery. The patient received adjuvant radiation therapy with 44 Gray in 22 fractions. Follow-up MRI showed no evidence of recurrence 5 years after surgery and the patient remains now in complete remission, fully self-dependent with only mild sensory deficits at lower extremities (Additional file 1).

## Discussion and conclusions

First described by Lichtenstein and Bernestein in 1959, MCS is a malignant tumor arising from bone or soft tissues [5]. Dowling reported the first case of MCS in non-osseous tissues in 1964 [6]. To date, an increasing number of reports suggest that MCS can occur anywhere in the body and at any age, with a male-to-female ratio of 1:1 and approximately 70% of the cases occurring during the second and the third decades of life [7].

Sparse cases of intradural MCS had been reported but there is not yet a systemic review in the literature. Compared to MCS at other anatomic sites, intradural MCS possesses a rather different clinical feature. Including the current case, the clinical information for 18 patients with primary intradural MCS in the literature is summarized in Table 1 [2, 8–21]. The current case is the eldest patient diagnosed with primary intraspinal MCS. Of all reported cases with primary intradural MCS, two-thirds of these patients were younger than 20 years old at the time of diagnosis. The male-to-female ratio was 1:1. The most prevalent symptoms were back pain and radicular pain, followed by sensory deficits, and muscle weakness. It was rare that urinary difficulty and Brown-Séquard syndrome were found as the initial manifestation. 50% (9/18) of intradural MCSs located in the thoracic spine, 17% (3/18) in the lumbar spine, 11% (2/18) in the cervical spine, 11% (2/18) in the thoracolumbar junction, and 11% (2/18) with disseminated lesions. As for the treatment, two-thirds of patients had gross tumor resection (12/18) and half of patients had adjuvant therapies (9/18). Only 1 case of local recurrence was reported during follow-up period (Case #16) and there was only 1 case of mortality (Case #10).

**Table 1:** Clinical features of primary intradural MCS published from 1978 to 2019

Case	Author	Age (yrs)/Sex	Symptoms (duration)	Tumor level	Tumor size (cm)	Dural attachment	Treatment	Recurrence	Outcome (FU from DG)
1	Scheithauer et al. [8]	5/M	N/A	L2-L4	N/A	(+)	R (NS)	No	Alive (2 yr)
2	Scheithauer et al. [8]	7/M	N/A	T10	1	(+)	R (NS)	No	Alive (3 yr)
3	Scheithauer et al. [8]	15/F	N/A	T9-T10	N/A	(+)	R (NS)	No	Alive (2 yr)
4	Lee et al. [9]	18/F	BP, RP, SD, MW, BSS (8 months)	T5-T6	N/A	(+)	GTR/RT	No	Alive (3 yr)
5	Huckabee et al. [10]	7/F	BP, RP (8 months)	L3	3 × 2	(+)	GTR	N/A	N/A
6	Ranjan et al. [11]	52/M	RP, SD, MW, UD (1 year)	C3-C6	N/A	(−)	GTR	No	Alive (6 mo)
7	Rushing et al. [12]	19/M	N/A	T5-T10	N/A	(+)	GTR/RT	No	Alive (14 yr)
8	Li et al. [13]	3/F	RP, SD, MW (10 months)	T11-L1	3 × 2 × 2	(−)	GTR/RT	No	Alive (2 mo)
9	Belhachmi et al. [14]	13/F	BP, RP, SD, MW (2 months)	T7-T8	N/A	(+)	GTR	No	Alive (2 yr)
10	Sharma et al. [15]	46/M	SD, MW, UD (15 days)	Disseminated	Disseminated	(−)	R/RT	N/A	Died**
11	Turel et al. [16]	6/M	BP, MW (4 months)	T9	2	(+)	GTR	N/A	N/A
12	Lee et al. [17]	17/M	BP, RP	Disseminated	Disseminated	N/A	R/RT/CT	N/A	N/A
13	Anderson et al. [18]	10/F	BP (9 months)	T4	1.5	(−)	GTR/RT	No	Alive (2 yr)
14	Yang et al. [19]	33/F	BP, RP, SD (5 months)	L1-L2	N/A	(+)	GTR*	No	Alive (3 yr)
15	Di Giannatale et al. [20]	14/M	BP, RP, SD (2 weeks)	T11-T12	2.2	(+)	GTR	No	Alive (2 yr)
16	Derenda et al. [2]	22/F	SD, RP, UD (2 months)	T12-L1	2.2 × 1.9 × 1.2	(−)	GTR/RT/CT	LR^*^	Alive (14 yr)
17	Presutto et al. [21]	21/M	NP, RP, SD, MW (3 months)	C2-C3	1.4 × 1.7 × 1.2	N/A	R/RT/CT	No	Alive (2 yr)
18	Current case	64/M	BP, SD, MW, UD, BSS (1 month)	T3	2 × 1.5	(+)	GTR/RT	No	Alive (5 yr)

*FU* follow up, *DG* diagnosis, *N/A* limited information, *BP* back pain, *RP* radicular pain, *NP* neck pain, *SD* sensory deficit, *MW* muscle weakness, *UD* urinary difficulty, *BSS* BrownSéquard syndrome, *R* resection, *GTR* gross tumor resection, *R (NS)* R/GTR (not mentioned specifically), *RT* radiotherapy, *CT* chemotherapy

LR* = local recurrence was noted at 4, 6, and 10 years after the initial resection, respectively

GTR*: post-op RT was suggested but patient refused due to financial concern

Died**: the patient died at non-specific timing before scheduled adjuvant chemotherapy

Histologically, most MCSs exhibit a biphasic pattern of islands of cartilage and areas of neoplastic small blue round cell component [5]. The exact histogenesis of intradural chondrosarcomas is still arguable. A probable hypothesis states that chondrosarcomas originate from primitive multipotential mesenchymal cells [22]. From the perspective of molecular pathology, the fusion gene encoding for the transcript *HEY1-NCOA2* had been discovered in 2012 and have become a powerful tool for diagnosis. It showed both high sensitivity and high specificity since *HEY1-NCOA2* was detected in nearly all cases of MCSs but not in other types of chondrosarcoma or Ewing sarcoma [3].

MRI remains the preferred imaging modality for intraspinal tumors, but there is no pathognomonic description for extra-osseous MCS. However, extra-osseous MCS typically present isointense signals with respect to the normal spinal cord on T1- weighted images while T2-weighted images show a high intensity or isointensity [23]. Besides, meningioma typically presents on MRI as a well-defined mass that is isointense to gray matter on T1-weighted images demonstrating avid enhancement after gadolinium administration, as in our case, making the differential diagnosis even more challenging [21]. Calcification can be seen occasionally but is believed to be not significantly related to the histologic findings and prognosis [23].

Radical surgery with complete removal of the tumor is considered the best choice of therapy for intradural MCS [13]. Clear resection margin predicts fewer local recurrences [24]. Due to the rarity of MCS, especially located in the intradural space, there is no general agreement on the necessity of adjuvant radiotherapy or chemotherapy. However, it has been demonstrated that adjuvant radiotherapy may reduce local recurrence [25] and chemotherapy may be associated with fewer recurrences especially for localized tumors [24]. From the aspect of intradural extramedullary tumors, even if total tumor resection is achieved during surgery, radiotherapy is indicated for intradural malignant tumors and chemotherapy is reserved for recurrent tumors with no other options in adult patients [4]. The dosage of post-operative radiation therapy was 44–78 Gray in previous studies [25]. The current case had adjuvant radiation therapy with 44 Gray in 22 fractions after the surgery of total tumor resection.

In general, the prognosis of MCS is poor regardless of the primary site of occurrence and 10-year survival rates in the literatures varying from 21 to 67% [26]. Local recurrence or distant metastases of MCS, particularly to lungs, lymph nodes and other bones, may appear even many years after the initial treatment [12]. Therefore, long-term follow-up is mandatory. In contrast to classic MCS, the prognosis of intradural extramedullary spinal MCS is remarkably better with only 1 case of local recurrence and 1 case of mortality among 18 cases reported in the literature. The superior treatment results of intradural extramedullary MCS maybe because complete resection of intradural extramedullary tumors is usually achievable and the neurologic prognosis after total tumor resection is often better than the other spinal neoplasia [4]. Some authors suggested that intraspinal MCS with dural attachment appeared to have a more favorable prognosis in comparison with those at other locations. It would be a result of early diagnosis and surgical intervention since neurologic deficit due to spinal cord compression would have already been noticed when the tumor is still small [7, 12].

In conclusion, MCS rarely located inside the dura sac of the spine. It occurs more frequently in pediatric patients and less in adults. In contrast to classic MCS, the functional recovery of intradural extramedullary spinal MCS is usually excellent as total tumor resection is commonly achievable. Adjuvant radiotherapy may reduce local recurrence and chemotherapy may be associated with fewer recurrences especially for unresectable tumors.

## Additional file

Additional file 1: Clinical timeline of our patient. (PDF 27 kb)

## Data Availability

Data sharing is not applicable to this case report as no datasets were generated or analyzed during the current study.

## Abbreviation

MCS: Mesenchymal chondrosarcoma
