# Supplementary figures and images for: Primary intradural extramedullary spinal mesenchymal chondrosarcoma: case report and literature review
Source: BMC Musculoskelet Disord. 2019 Sep 4;20:408. doi: 10.1186/s12891-019-2799-2 (PMC6727557; doi:10.1186/s12891-019-2799-2)

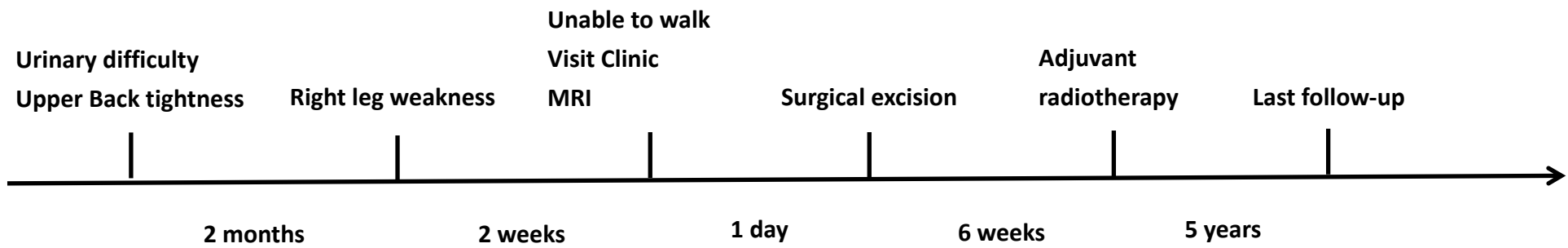

Supplement: Supplementary file 1 — Clinical timeline of our patient. (PDF 27 kb) [file 12891_2019_2799_MOESM1_ESM.pdf]
